# Bringing asthma care into the twenty-first century

**DOI:** 10.1038/s41533-020-0182-2

**Published:** 2020-06-05

**Authors:** Kjell Larsson, Hannu Kankaanranta, Christer Janson, Lauri Lehtimäki, Björn Ställberg, Anders Løkke, Kristian Høines, Klaus Roslind, Charlotte Suppli Ulrik

**Affiliations:** 1 Integrative Toxicology, National Institute of Environmental Medicine, IMM, Karolinska Institute, Stockholm, Sweden; 2 Department of Respiratory Medicine, Seinäjoki Central Hospital, Seinäjoki, Finland; 3 Faculty of Medicine and Health Technology, University of Tampere, Tampere, Finland; 4 Department of Medical Sciences: Respiratory, Allergy and Sleep Research, Uppsala University, Uppsala, Sweden; 5 Allergy Centre, Tampere University Hospital, Tampere, Finland; 6 Department of Public Health and Caring Sciences, Family Medicine and Preventive Medicine, Uppsala University, Uppsala, Sweden; 7 Department of Medicine, Little Belt Hospital, Vejle, Denmark; 8 Tananger Medical Center, Tananger, Norway; 9 Aarup Doctors Medical Centre, Aarup, Denmark; 10 Respiratory Research Unit Hvidovre, Department of Respiratory Medicine, Copenhagen University Hospital Hvidovre, Hvidovre, Denmark; 11 Institute of Clinical Medicine, University of Copenhagen, Copenhagen, Denmark

**Keywords:** Patient education, Disease prevention

## Abstract

Despite access to diagnostic tests and effective therapies, asthma often remains misdiagnosed and/or poorly controlled or uncontrolled. In this review, we address the key issues of asthma diagnosis and management, recent evidence for levels of asthma control, the consequences of poor control and, in line with that, explore the potential reasons for poor asthma control and acute exacerbations. Based on recent evidence and current guidelines, we also aim to provide practical answers to the key questions of how to improve asthma management, with the best possible prevention of exacerbations, addressing the basics—adherence, inhaler misuse, obesity and smoking—and how to facilitate a new era of asthma care in the twenty-first century. We hope this review will be useful to busy primary care clinicians in their future interactions with their patients with both suspected and proven asthma.

## Introduction

**Fig. 1:**
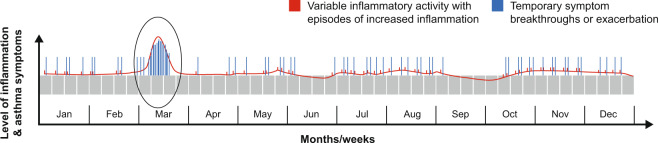
Variability of inflammation and symptoms. Hypothetical illustration of asthma: a disease of chronic inflammation, with episodes of worsening inflammation associated with increased (breakthrough) symptoms and/or exacerbations.

Our current understanding is that asthma is a common and potentially life-threatening chronic inflammatory airway disease, with different phenotypes, characterised by variable airflow obstruction and, even in mild cases, with unpredictable, recurrent episodes of worsening symptoms^1,2^. Typical symptoms include wheeze, cough, shortness of breath and chest tightness that can vary in intensity over time, spontaneously or with pharmacological treatment^1^. Periods of symptom breakthroughs, commonly due to fluctuating inflammatory activity, can develop into exacerbations that may require urgent healthcare and, in some cases, may even be fatal^1–6^ (Fig. 1). Although exacerbations are more common and of greater severity in patients whose asthma is poorly controlled or more severe^1,7^, even patients with mild asthma are at risk of breakthrough symptoms and exacerbations^1,2,8^.

Inhaled corticosteroids (ICS) were first introduced as an anti-inflammatory treatment in the 1970s^9,10^, and despite subsequent advances in our understanding of asthma and its various phenotypes, new medications and inhaler devices, and evidence-based management guidelines, asthma-related morbidity (i.e. uncontrolled asthma and exacerbations) are still a widespread problem although mortality rates have declined^1,2,7,9^.

In this review, we aim to address key issues, review recent evidence and provide practical answers to the question of how we can optimise current management of asthma and bring the care of this common disease into the twenty-first century.

## What are the goals of diagnosis and treatment in asthma?

Correct diagnosis is essential to ensure that every patient receives treatment appropriate to their condition and, unfortunately, misdiagnosis of asthma is still common^11^. Correct diagnosis of asthma is generally based on a history of symptoms, family history (e.g. atopic disease), physical examination and, as the essential part of the diagnostic process, demonstration of variable airflow limitation by spirometry or peak flow measurement, with consideration of differential diagnoses^12,13^ (Table 1).

**Table 1:** Factors in asthma diagnosis in adults^18^.

Parameter	Details
Spirometry: forced expiratory volume in 1 s (FEV_1_)	Increase in FEV_1_ of >12% and >200 mL after inhaling a bronchodilator (greater confidence if increase is >15% and >400 mL)
Peak expiratory flow (PEF)	Excessive (>10%) variability in daily diurnal peak expiratory flow (PEF)
Methacholine, mannitol or adenosine 5′-monophosphate (AMP) challenge	Direct and indirect bronchial challenges can help to confirm the diagnosis of asthma
Differential diagnoses for patients presenting with wheeze and/or breathlessness, without an obvious history of asthma	These include chronic bronchitis, heart failure, pulmonary emboli, dysfunctional breathing, laryngeal obstruction, and central airway tumours. Patients with COPD or asthma with concomitant COPD may also present with bronchodilator reversibility and/or PEF variability^109,110^. In older patients with a history of smoking or other harmful environmental exposures, COPD or asthma/COPD overlap may be considered.

**Fig. 2:**
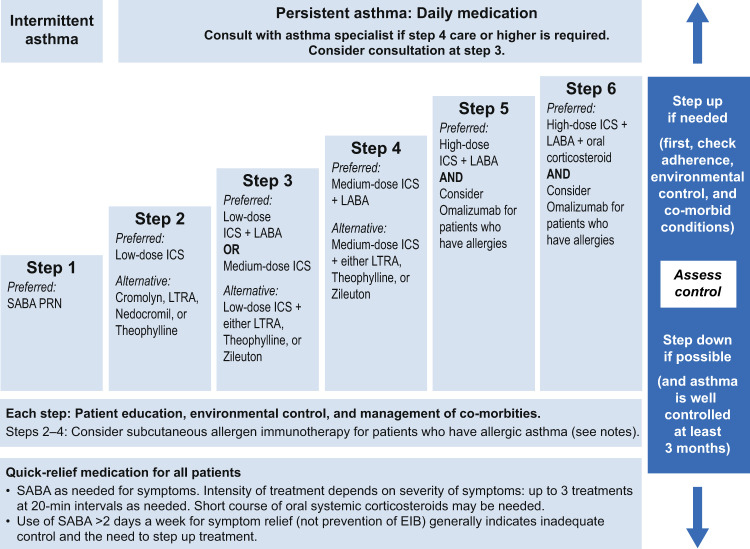
Stepwise approach for managing asthma in adolescents and adults. Available at https://www.ncbi.nlm.nih.gov/books/NBK7222/figure/A2212/ (accessed 20 January 2020). EIB exercise-induced bronchospasm, ICS inhaled corticosteroid, LABA long-acting inhaled beta2-agonist, LTRA leukotriene receptor antagonist, SABA short-acting beta2-agonist. Source: National Heart, Lung, and Blood Institute; National Institutes of Health; U.S. Department of Health and Human Services.

Various guidelines and reports, such as the GINA Global Strategy for Asthma Management and Prevention, have been developed with the aim of providing consistency of asthma treatment around the world^1,3,4,13–15^. Most guidelines share a ‘step care’ approach to treatment, with the aim of achieving daily asthma control and preventing exacerbations (future risk) using the lowest level of medication needed to achieve these goals (Fig. 2). Controller medication should be stepped up or down in line with the observed variations in level of asthma control which can be detected by regular assessment, treatment and review^1,3,5,13,14^.

**Fig. 3:**
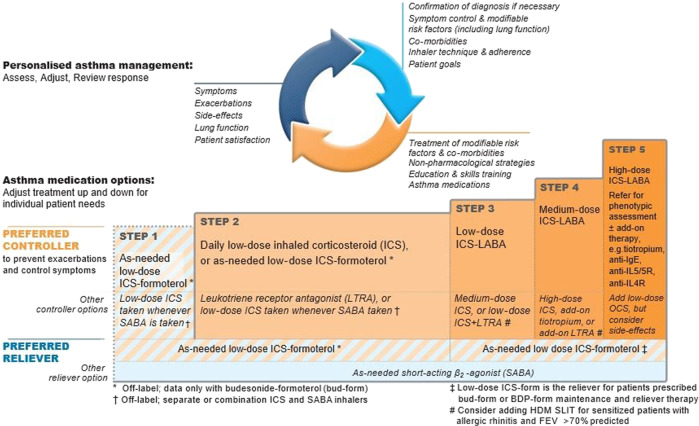
The GINA 2019 asthma treatment strategy for adults and adolescents ≥ 12 years. Box 3−5A. Available at https://ginasthma.org/wp-content/uploads/2019/06/GINA-2019-main-report-June-2019-wms.pdf (accessed 20 January 2020). © 2019 Global Strategy for Asthma Management and Prevention, all rights reserved. Use is by express license from the owner.

For many years, the lowest treatment step, recommended in most guidelines for intermittent or mild asthma, has been a short-acting β_2_-agonist (SABA) reliever, which relieves bronchoconstriction rapidly and effectively but does not reduce the underlying inflammation usually present even in mild asthma^16,17^. The recommendation for SABA alone as initial treatment for mild asthma dates back to the era when asthma was thought to be a disease only of bronchoconstriction^10,18^. Also, the development of SABA predated the development of ICS by many years, so SABA use became ingrained in the management of asthma^10^. Over-reliance on β_2_-agonist bronchodilators may even worsen inflammation and increase the risk of exacerbations and hospital admissions^1,19–23^. The 2019 update to the GINA guideline now recommends replacing SABA with low-dose ICS/formoterol as preferred reliever, for safety reasons, both for mild asthma and also at higher GINA steps, in patients already on ICS/formoterol maintenance therapy^18^ (Fig. 3).

Anti-inflammatory therapy with ICS is recommended as maintenance therapy, initially at a low dose but at higher doses for more severe asthma^1,3,5^. However, in patients on ICS, add-on of a long-acting β_2_-agonist (LABA) has been shown to be more effective than increasing the ICS dose in improving asthma control and preventing exacerbations^24,25^. As a result, an ICS/LABA combination inhaler is now the first choice of maintenance therapy for the majority of patients with moderate-to-severe asthma^1^.

Other treatment options include leukotriene receptor antagonists (LTRA), which have a less potent anti-inflammatory activity than ICS, short- or long-acting muscarinic antagonists (SAMA or LAMA) as alternative or additional bronchodilator relievers, and the recently developed injectable biologic drugs for patients with specific subtypes of severe asthma^1,26^.

## What is meant by asthma control?

Well-controlled asthma means that patients are free from troublesome respiratory symptoms during both day and night, need little or no reliever medication (no more than two puffs SABA/week), can lead normal, productive and active lives and continue to have normal or the best possible lung function^1,3,5,13^. Daytime symptoms or use of reliever more than twice a week, night-time awakenings or limitation of activity all suggest only partial control and if a patient is experiencing all of these then their asthma can be considered uncontrolled^1,18^.

Achieving well-controlled asthma greatly reduces, but does not eliminate, the risk of breakthrough symptoms and exacerbations of asthma resulting from increases in the airway inflammation that underlies most patients’ asthma^27^. Even patients with mild or intermittent asthma are at risk of these exacerbations^6,17^. However, in many cases there is a discrepancy between what patients and healthcare professionals understand by the term ‘asthma control’^8^. For many patients, ‘control’ simply means being able to keep their symptoms at a manageable level through frequent use of reliever medication^28,29^. For healthcare professionals, the definition of asthma control is usually broadly based on guideline definitions—absence of symptoms and restrictions on daily activities, good lung function with minimal or no use of reliever and no sleep disturbances^5,18^.

## How well are patients achieving asthma control?

**Fig. 4:**
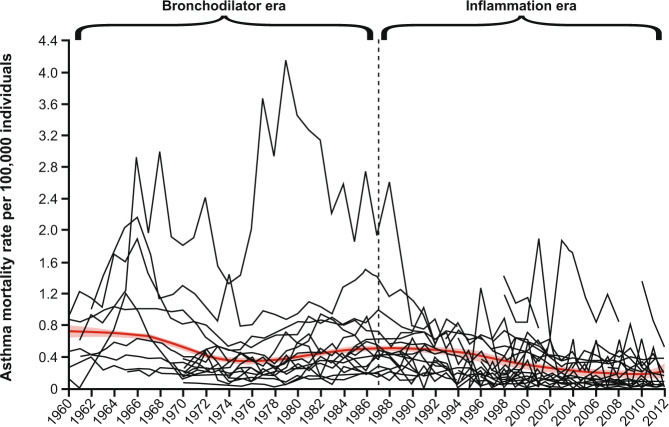
Asthma mortality over time. Crude asthma mortality rates during the bronchodilator and anti-inflammatory eras. Reproduced from Pavord et al.^2^.

Given the availability of evidence-based reports and guidelines, along with a range of effective medications and inhaler devices to deliver those medications to the target tissues, most patients nowadays should have well-controlled asthma. Regrettably, however, although hospital admissions and asthma mortality have decreased over recent decades, rates now appear to have plateaued^2,7,9,30–33^ (Fig. 4). Several real-world surveys have indicated that, at best, only 50% of patients with asthma meet the criteria for well-controlled asthma, indicating either that these criteria are too strict or that asthma management is inadequate^12,28,29,34–36^.

**Fig. 5:**
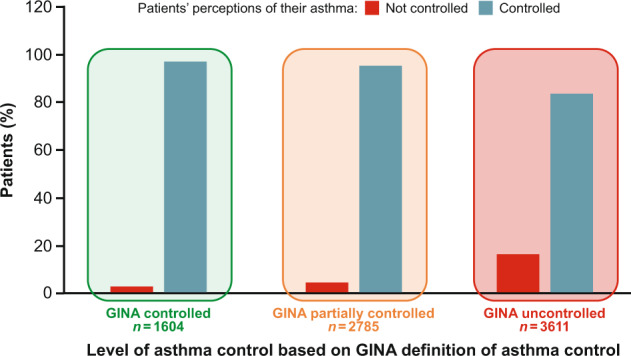
Asthma control and patient perception. The mismatch between patient perceptions of their asthma control and objective assessments. Source: Price et al.^29^.

A range of tools has been validated for the assessment of asthma control, including the Asthma Control Questionnaire (ACQ) and Asthma Control Test™ (ACT)^37^. In the INSPIRE study (*n* = 3415), comparing patients’ own assessment of their level of control with ACQ scores, most patients (89%) had experienced a mean of 12 periods of symptom worsening within the previous year, despite reporting that they believed their asthma was controlled or even well-controlled^28^. Even the 28% with asthma classed objectively as well-controlled reported an average of 6.3 asthma worsenings a year^28^. A study by Haughney et al.^38^ found that 91% (*n* = 468) of respondents felt that their asthma was under control, yet two-thirds (*n* = 339) experienced symptoms at least 2–3 times a week. Similarly, in the REALISE study (*n* = 8000), among patients with GINA-defined partially controlled and uncontrolled asthma, 95% and 84% respectively stated that they had controlled asthma, despite the fact that 55% reported that their daily life was affected by their asthma and 52% had been awakened at least once in the previous week^29^ (Fig. 5).

Few patients are aware of the treatment goals outlined in the guidelines^38^. A UK-wide study showed that 58% of patients were initially satisfied with the standard of their asthma management and control^38^. However, after being shown international asthma guidelines on the outcomes they should expect from their treatment, this declined to only 33%^38^.

## What are the consequences of poor asthma control?

In addition to respiratory problems, poorly controlled asthma has been shown to reduce the general health-related quality of life and affect several aspects of human life such as mobility, sleeping, everyday activities, mental function, discomfort, depression, distress, vitality and sexual activity^39^. Poor asthma control causes symptoms affecting daily activities, well-being and quality of sleep patterns of patients, all having a negative impact on quality of life^40^. Poor asthma control also increases the risk of asthma deteriorating into acute exacerbations^41^. The most important risk factors for exacerbations are having uncontrolled asthma, a history of previous exacerbations and/or hospitalisation, over-reliance on SABA, elevated blood eosinophils and respiratory viral infections^41–43^. In a UK National Health & Wellness Survey of patients being treated with ICS/LABA, compared with well-controlled disease, poorly controlled asthma was associated with more emergency department visits (21% vs. 14%; *p* = 0.016) or hospitalisations (13% vs. 8%; *p* = 0.022) in the previous 6 months, lower mental and physical health-related quality of life (*p* < 0.001) and impaired work productivity (29% vs. 17%; *p* < 0.001) and activity scores (46% vs. 24%; *p* < 0.001)^44^. Over 70% of the patients in the INSPIRE study also reported that one of the worst things about having asthma was the panic they felt when their symptoms worsened^28^.

When patients have asthma exacerbations, they are likely to receive treatment with oral corticosteroids (OCS)^45^. Almost half of the respondents in the REALISE study reported that they had acute exacerbations requiring OCS for asthma in the previous year and almost one-quarter had visited the emergency department^29^. In a Swedish study, 22.5% of patients with asthma (*n* = 49,930) were periodic users of OCS (>0 but <5 mg/day/year) and 1.5% (*n* = 3299) were regular users (≥5 mg/day/year). The percentage of patients in REALISE who had an acute exacerbation resulting in OCS treatment in the previous year ranged from 26% to 29% for those with mild asthma (GINA steps 1−2) to 61% for those with more severe asthma (GINA step 4)^29^. Minimising exposure to OCS by improving asthma control is important as repeated or maintenance treatment with OCS increases the risk of adverse effects such as development of osteoporosis, peptic ulcer, diabetes, cataracts and fractures^45–47^.

## Why is asthma control poor and why do exacerbations occur?

The reasons for poor asthma control can be divided into three categories: patient-related, healthcare-related, and therapy-related^48^ (Table 2). The most important of the patient-related reasons for poor asthma control in the twenty-first century include obesity, tobacco smoking, over-reliance on reliever therapy and underuse of maintenance controller medication. The inability to use inhalers correctly, and poor perception of asthma symptoms also contribute to poor control^1,12,43,49,50^. At times of worsening symptoms, most patients increase their SABA use early and many only increase their ICS or ICS/LABA later when symptoms are at their worst^28^. Using only SABA during symptom breakthroughs is a paradoxical approach since SABA alone does not address the increased inflammation during occasional episodes in response to trigger factors such as exercise, cold air and aeroallergens^1,8,51–54^. The pathophysiological changes in response to a trigger factor result in an inflammatory flare-up and release of a wide variety of inflammatory mediators within the airways^5^. Regardless of the trigger, there is a rapid smooth muscle contraction, mucosal oedema and mucus hypersecretion which together lead to the development of airway obstruction and symptoms.

**Table 2:** Factors involved in poor asthma control.

Patient-related	Healthcare-related	Therapy-related
•Poor adherence ♦Unintentional (forgetting to take the medicine) ♦Intentional (asthma ‘feels OK’—stops taking the medication) ♦Fear of corticosteroid side effects •Smoking •Inhalation-related errors ♦Inability to use inhaler correctly ♦Incorrect handling of the inhaler ♦Wrong/poor inhalation technique •Poor perception (don’t notice a deterioration) •Lack of self-management plan •Adjusting medication incorrectly at times of asthma worsening ♦Increasing number of SABA inhalations instead of ICS-containing drugs	•Underestimate of asthma severity •Lack of asthma reviews (asthma assessment/annual reviews) •Prescription renewals via email/phone without either asthma assessment or device handling and inhalation technique •Incorrect or insufficient treatment—the right dose of the right drug in the right inhaler needs to be chosen for the individual patient	•The SABA paradox—treating symptom breakthroughs with SABA only, so not treating any underlying increase in inflammation

**Fig. 6:**
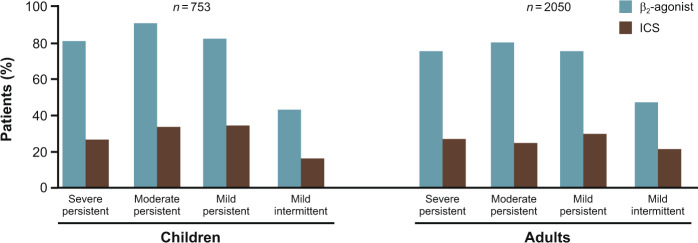
Use of drugs in asthma. Reliance on SABA and underuse of anti-inflammatory treatment in the AIRE survey. Adapted from Rabe et al.^31^.

Many patients with poorly controlled asthma are over-reliant on their SABA for relief of symptoms^28,29,31,35^. They feel rapid symptom relief every time they use the SABA, whereas they feel no immediate benefit from inhaling ICS. This is often the reason for poor adherence to their ICS-based maintenance regimen^8^. In the seven-country AIRE study, SABA use was ~3 times greater than ICS use over a 4-week period, and in Italy and France recent ICS use was reported by less than one in nine patients who reported recent use of SABA^35^. Other studies show a similar imbalance in the ratio of ICS maintenance to SABA reliever treatment use (Fig. 6).

Overall, maintenance medication adherence rates in patients with asthma have consistently been shown to be around 30–40% in practice, with a systematic review finding that 24% of exacerbations and 60% of asthma-related hospitalisations could be attributed to poor adherence to ICS^55,56^. In the REALISE study, some 52% of patients prescribed daily anti-inflammatory maintenance treatment (*n* = 3481) did not take this medication daily^29^. The INSPIRE study gave similar results, with 50% of patients saying they adjusted how much ICS/LABA they took according to how they felt, 25% of patients stating that they did not feel they needed to take their maintenance therapy everyday when they felt well and 74% having used their SABA everyday in the previous week.

Possibly because of inadequate asthma education/knowledge and lack of advice and follow-up, many patients do not understand that the need for reliever use is a sign of deteriorating asthma and that they need instead to increase their anti-inflammatory controller medication^2,29^. Use of SABA alone can mask increasing inflammation until it reaches a level that requires urgent medical attention. Regular or frequent use of β_2_-agonists is associated with adverse effects including β-receptor downregulation^57^, reduced bronchoprotection against constrictor stimuli^58^, rebound bronchial hyperresponsiveness and reduced bronchodilator response to β-agonist during acute bronchoconstriction^57^. Indeed, it has been shown that SABA used alone induces production of the proinflammatory cytokine IL-6 and this production is significantly augmented during virus infection^59^. Concurrent treatment with ICS reduces these adverse effects^59–61^.

Using ≥3 ×200-dose canisters of SABA a year was associated with double the risk of an asthma-related exacerbation in one study^20^. Every additional SABA canister was associated with an 8–14% and 14–18% increase in risk of an asthma-related exacerbation in children and adults, respectively^20^. Multivariate analyses in adults (*n* = 35,864) also showed that the risk of hospitalisation was significantly associated with prescription of SABA inhalers above a baseline of 1–3 per year (4–12 SABA: OR 1.71; 95% CI 1.20–2.46)^22^. In a Canadian database analysis (*n* = 343,520), inappropriate use of SABAs in any 1-year period was associated with a 45% (OR 1.45, 95% CI 1.26-1.66) increase in the risk of asthma-related admissions in the following 3-month period^23^. An earlier study had demonstrated that dispensing of ≥2 SABA canisters a month was associated with increased risk of death^62^. The UK Royal College of Physicians in their recent National Review of Asthma Deaths found a similar association between risk of death from asthma and prescription of ≥12 SABA inhalers per year^6^. They also found evidence of previous under-prescribing of preventer ICS medication for those patients who died^6^.

This reliance on SABA treatment is reinforced by its rapid relief of symptoms, its prominence in emergency primary care and hospital management of exacerbations, and, in many countries, its low cost and availability. Repeat prescriptions for SABA may be given through online systems, email or by telephone, and in some countries patients can get their SABA ‘over the counter’. Consequently, patients may not have their use of medication reviewed or their asthma control re-assessed.

## How can we improve asthma control and prevent exacerbations?

Approaches to improving asthma control include patient and HCP education, regular review and assessment of asthma status and inhaler technique along with use of a wide range of interventions and technologies to try to improve adherence to inhaled asthma medications^63^. These include enhancing communication skills, structured frameworks such as SIMPLES (Smoking status, Inhaler technique, Monitoring, Pharmacotherapy, Lifestyle, Education, Support) and various forms of electronic monitoring, with or without reminders for when to take controller medication^63,64^.

### Improving adherence

A recent Cochrane review included 28 studies regarding a range of interventions to improve adherence to ICS maintenance therapy for asthma^65^. The authors concluded that patient education, electronic trackers or reminders and simplified regimens generally improved adherence but did not consistently translate into observable benefit for clinical outcomes^65^. In a study by Foster et al.^66^, despite personalised adherence discussions or inhaler reminders and feedback in connection with prescribed fixed combination ICS/LABA controller therapy, adherence decreased over 6 months to as low as ~38% with personalised advice whilst electronically measured adherence decreased to 60% even in patients given inhaler reminders and feedback. Even after potentially life-threatening emergency department visits, adherence to ICS maintenance decreased to 50% within the first week after discharge^67^.

### Asthma action plans

Optimal self-management involving provision of a written asthma action plan was shown to reduce unscheduled primary care visits and hospitalisations in a Cochrane review^68^. However, education alone was not included in the analysis as previous work had shown that without an action plan, self-monitoring or regular review, information-only education had no significant impact on objective health outcomes^68^. All asthmatics should be offered a self-management action plan that advises them how to recognise and respond to a deterioration in their level of asthma control^69^. Self-management plans that involve patients doubling their ICS dose when symptoms worsen do not appear to be fully effective in preventing exacerbations^70,71^, although it has recently been shown that a temporary four-fold increase in ICS had some beneficial effects^72^.

### Regular asthma assessment

Regular systematic asthma reviews, at least once a year, have been shown to help improve asthma control and reduce exacerbations^6,18^. Reviews are an ideal opportunity for the practitioner to check the patient’s inhaler technique, discuss adherence to maintenance therapy, reinforce patient education on asthma and its treatment and, for smokers, to discuss the benefits of cessation^18^ (Table 3). In an observational study (PACEHR) of 18,724 patients with asthma in Sweden, 96% had mild-to-moderate asthma and 4% had severe asthma requiring high-dose ICS and a second controller. Only a minority of patients had their asthma assessed in the year prior to the index date, and of the patients with severe asthma, only one in five had visited a specialist in secondary care in the year before and after an index date^73^. Many patients do not visit their primary care doctor or nurse for routine asthma reassessments and rely on their SABA to manage symptoms as they occur^28,29^.

**Table 3:** Checklist: Practical points for achieving and maintaining asthma control for your patients.

Basic skills needed for asthma reviews	Comments
Know what is meant by well-controlled asthma	•Patients should be free from troublesome respiratory symptoms during both day and night, need little or no reliever medication (not more than two puffs of SABA/week), can lead normal, productive and active lives and continue to have normal or best possible lung function.
Know the cut-off points for controlled, not well-controlled and uncontrolled asthma	•GINA parameters—Not well-controlled: one or two of daytime symptoms or use of reliever more than twice a week, any night-time awakenings or limitation of activity; Uncontrolled: three or all four are present
Know how to measure asthma control by questionnaires	•Use validated questionnaires such as Asthma Control Questionnaire (ACQ) or Asthma Control Test (ACT)
Knowledge of spirometry and how to assess results	•Have access to spirometer and/or peak flow metre. Undergo training if required
Knowledge about the most commonly used inhalers	•See the guidance from the ADMIT group, available at: https://www.inhalers4u.org/index.php/instructions/
Knowledge of correct use of inhalers	•Inhaler usage instruction videos available in four languages at: www.inhalatorgebruik.nl

At every asthma review visit	Comments
History—assess symptoms and SABA use in past weeks	•Symptoms should be minimal, SABA use less than two occasions/week, no nocturnal symptoms
Assess asthma control by validated tool	•Validated tools include Asthma Control Questionnaire (ACQ) and the Asthma Control Test™ (ACT)
Assess inhaler use and inhalation technique	•Ask patient to demonstrate inhaler technique
Consider risk of exacerbations	•Ask if symptoms have increased recently, beyond the normal day-to-day pattern
Spirometry or peak flow measurement	•Compare with normal values for age/height/weight
Check optimal use of maintenance medication (od or bid)	•Ask open questions like ‘How often do you take your medication’, rather than ‘Do you take it as I prescribed?’
Evaluate signs of difficult-to-treat or severe asthma	•Do symptoms persist despite adequate use of medication?
Consider need to confirm the diagnosis?	•Do symptoms persist despite adequate use of medication or has the patient had no symptoms for some time?
Make another follow-up appointment	

Face-to-face interactions with a general practitioner or asthma nurse during an asthma review can motivate improvements in adherence and enable the individual’s asthma severity and inhaler technique to be assessed^74^. Inadequate inhalation technique has been observed in up to 90% of patients and is associated with poor asthma control and more frequent visits to emergency departments^49,75,76^. Even after training, 50% of people with poor technique revert to their old habits or develop new errors over time^49^, emphasising the need for regular checks on inhalation technique to avoid ineffective treatment and waste of medication^1,3,49^. During a review, asthma guidelines recommend asking about asthma symptom breakthroughs and SABA use/week, night-time awakening/coughing or exacerbations, testing lung function and using an objective assessment tool such as the ACT or ACQ.

Mental health, co-morbidities, poverty, drug abuse, financial hardship, poor literacy, pet ownership and many other personal factors can also affect a patient’s self-management of asthma and are factors that could potentially be detected and addressed during face-to-face interviews with patients^77,78^.

### Smoking cessation and weight loss

Asthma reviews are also an opportunity for the clinician to recommend smoking cessation to patients who continue to smoke tobacco, offering treatment if the patient agrees. Patients who smoke should be told that due to smoking, their asthma control is worse, lung function decline is faster and they have a higher risk for hospitalisation^79,80^. The permeability of airway mucosa is increased in smokers, which could increase clearance of ICS from the airways^81^. Smoking also decreases histone deacetylase activity, which can reduce the ability of ICS to suppress inflammatory cytokine production (steroid resistance)^81^. Similarly, advice and recommendations on weight loss can be provided to patients with a high BMI, as this, like smoking, is a significant risk factor for poor asthma control and exacerbations^43^ and studies have shown that a 5–10% reduction in body weight improves asthma control and lung function^82,83^.

### Exposure avoidance

Avoiding or minimising exposure to allergens and environmental irritants/pollutants can help patients with allergic or occupational asthma, although few studies have shown significant results from allergen avoidance alone^84^. However, recent studies using an overhead cooled laminar airflow filter device to displace aeroallergens from the breathing zone overnight in patient’s bedrooms improved quality of life and reduced airway inflammation (FeNO) and markers of systemic allergy (IgE and eosinophils) in patients with persistent atopic asthma^84,85^. Such devices are a new form of non-digital technology that may benefit patients with asthma in future.

## How can we bring asthma care into the twenty-first century?

### Digital technology

A growing number of asthma Apps are being developed for use on smartphones and other electronic devices^86,87^. These have the potential to aid self-management and to serve as useful tools in the patient−doctor relationship^88^. They can track the individual patient’s use of treatment and peak flow readings, provide dose reminders and help them to avoid exacerbation triggers such as high pollen counts or peaks in air pollution^86,89^. Some provide information on asthma, instructions and information on asthma medications, and what to do if symptoms worsen^87^.

Digitally enabled inhalers (so-called ‘smart inhalers’) are also becoming available, which can monitor medication use (time, date, number of inhalations) and, when connected wirelessly to a mobile phone, can send medication alerts/reminders for scheduled doses, which can improve both adherence and asthma-related health outcomes^90,91^. Data obtained from such devices can be used to deliver self-management interventions tailored to the specific needs of patients, thus increasing the efficacy of such interventions. Digitally enabled inhalers can also help to discriminate between patients with severe asthma and those who have poor inhaler technique and/or poor adherence^92^. These inhalers could help to identify patients with a genuine need for the newly available biologic therapies.

Portable spirometers and FeNO metres are also becoming more affordable and thus more widely available in primary care, enabling more accurate assessment of lung function and airway inflammation to be conducted by GPs or asthma nurses^93^.

### The GINA 2019 update

GINA describes this update as the biggest change to asthma management proposed in over 30 years. Single inhaler ICS/formoterol is now recommended as the preferred reliever in place of SABA alone across the full spectrum of asthma severity (only for patients already on ICS/formoterol maintenance at GINA steps 3–5)^18^ (Fig. 3). Recommending use of an anti-inflammatory combination reliever for this inflammatory disease, rather than SABA alone, which can worsen inflammation^8^, resolves a major paradox in most previous guidelines. The new approach proposed by GINA 2019 had already been suggested by a consortium of international experts on asthma management in the Lancet Commission 2017 ^2^. A large body of data already exists on the efficacy and safety of the budesonide/formoterol combination when used as an as-needed reliever medication in moderate-to-severe asthma^94–98^. There is also a study showing that symptom-driven use of beclometasone/salbutamol as reliever was as effective as regular use of beclometasone, with a lower cumulative ICS dose^99^. There are currently no data demonstrating the efficacy and safety of combining ICS/formoterol with maintenance ICS/LABA treatment that does not contain formoterol.

In mild asthma, as-needed use of budesonide/formoterol was shown in the recent SYGMA studies to be more effective and better tolerated than SABA alone^94,95,100,101^, and was clinically equivalent to daily maintenance therapy with budesonide with as-needed SABA as reliever, in terms of asthma control^94,95,101^. Use of as-needed budesonide/formoterol in SYGMA 1 reduced the rate of severe asthma exacerbations by 64% and the rate of moderate to severe exacerbations by 60% versus SABA alone while the severe exacerbation rates did not significantly differ between the as-needed budesonide/formoterol group and the budesonide maintenance group^94^. In the SYGMA 2 trial, as-needed budesonide/formoterol and maintenance budesonide were also equipotent in reducing the rate of severe exacerbations^95^. Importantly, however, the median daily doses of ICS were considerably lower with as-needed budesonide/formoterol than with daily maintenance therapy (metered dose, 57 vs. 340 μg and 66 vs. 267 μg, in SYGMA 1 and 2 respectively)^94,95^. The 52-week PRACTICAL and Novel START studies have confirmed these findings in a more pragmatic, real-world open label setting in which as-needed budesonide/formoterol was more effective at preventing severe asthma exacerbations than low-dose maintenance budesonide plus as-needed terbutaline, with a lower daily mean dose of budesonide (difference in PRACTICAL of 126.5 μg per day vs. maintenance; 95% CI −171.0 to −81.9; *p* < 0·001)^100,101^.

**Fig. 7:**
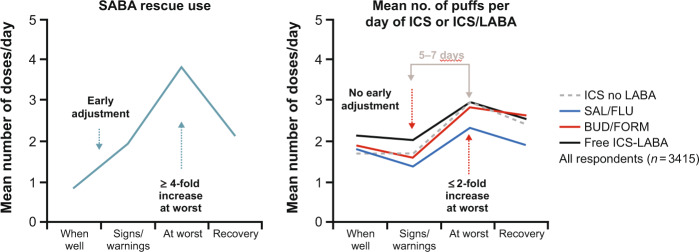
Typical patient behaviour. Increasing short-acting β_2_-agonist (SABA) use but not inhaled corticosteroids (ICS) use when symptoms worsen. Adapted from Partridge et al.^28^.

The period of worsening symptoms that usually precedes an exacerbation appears to be a ‘window of opportunity’, during which the extra doses of ICS provided by as-needed ICS/formoterol may be able to suppress the inflammatory flare-up and prevent the exacerbation from occurring or reduce its severity^28,102^. As-needed budesonide/formoterol has a significant advantage over as-needed SABA in that it provides the required immediate relief simultaneously with an anti-inflammatory boost of ICS during the ‘window of opportunity’^2,8,94^. Studies have shown that when symptoms appear or worsen, most patients instinctively reach for their SABA to relieve the symptoms and increase their use of this medication, rather than the controller needed to reduce the increased inflammation causing the worsening^2,8,28,29^ (Fig. 7).

### Add-on therapies

Another recent change to asthma management is the use of LAMA as an add-on to ICS or ICS/LABA maintenance therapy for moderate-to-severe asthma. Single inhaler ICS/LABA/LAMA combinations are already in development and will soon be available^103^. A systematic review comparing add-on LAMA with add-on LABA found that people taking LAMA + ICS scored slightly less well for quality of life and asthma control and had adverse events more often than those taking LABA + ICS^104^. As an add-on to ICS/LABA in patients with poorly controlled asthma, LAMA significantly increased the time to first exacerbation and gave a modest improvement in lung function in two randomised controlled trials with 912 patients^105^.

LRTAs are recommended in asthma guidelines as an alternative or add-on controller, but a systematic review concluded that ICS were more effective in both adults and children, particularly in patients with moderate airway obstruction^106^.

### Biologics

For patients with specific subtypes of severe asthma, such as eosinophilic asthma, a number of biologics have been developed for use when conventional therapy, and systematic assessment and optimising therapy of co-morbidities, does not lead to acceptable asthma control^26^. These include omalizumab, which targets IgE, mepolizumab, reslizumab, and benralizumab, which all target pathways to reduce eosinophil counts, and dupilumab which targets the interleukins IL-4 and IL-13. These are different approaches to reducing the underlying eosinophilic or Type 2 inflammation in asthma and have been shown to reduce exacerbation rates and improve asthma control^26^. All require injection, adding inconvenience to their already considerable costs, but for some patients they represent a real breakthrough in efficacy against their severe asthma, while avoiding exacerbations and hospitalisations can also make them cost-effective in the right patients^32^.

## Which patients should be referred to asthma specialists?

Patients with difficult-to-treat asthma should be systematically assessed to find out if they have a severe asthma or other reasons explaining their poor response to treatment, such as poor adherence or inappropriate treatment. For patients who do not respond to standard step care management and have poor asthma control despite good adherence and inhalation technique, including management of environmental exposures and co-morbidities, referral to a specialist is clearly essential^1,18,107,108^.

## Discussion

Despite improvements in understanding, availability of evidence-based management guidelines and improved medications and devices, approximately half of all patients with asthma are still not optimally controlled. A range of factors is responsible for this situation—the fluctuating nature of the disease, patients’ reluctance to take ‘steroids’ when feeling well, typical patient relief-seeking behaviour favouring SABA over ICS, the costs of medication, the different asthma phenotype responses to treatment, misperceptions of what asthma control means in practice and lack of interest in or knowledge of asthma among HCPs.

The twenty-first century now offers clinicians a range of new and different options to improve asthma control and help patients avoid exacerbations. Improved guidelines, electronic monitoring, smartphone apps, FeNO metres, portable spirometers, easy-to-use inhalers and, for patients with very severe asthma, biologic therapies, are all options that were unavailable to previous generations. Of course, regular review and assessment by knowledgeable physicians and specialist nurses, weight loss and smoking cessation will continue to play important roles, with or without these newer options.

However, the greatest impact on future care for the majority of patients, those with mild-to-moderate asthma, may come when the recommendations concerning ICS/formoterol as preferred reliever across the asthma severity spectrum are fully implemented. This would prevent over-reliance on SABA and ensure that patients receive a dose of anti-inflammatory ICS whenever they feel the need for additional relief of symptoms.
